# Respectful Patient–Provider Communication and Patient Portal Usage in Pregnant People of Color

**DOI:** 10.1089/heq.2024.0177

**Published:** 2025-06-16

**Authors:** Amy H. Goh, Christopher Lee, Chisolum Nkenke, Joyce K. Edmonds

**Affiliations:** ^1^ Thomas Jefferson University, Philadelphia, Pennsylvania, USA.; ^2^ William F. Connell School of Nursing, Boston College, Chestnut Hill, Massachusetts, USA.; ^3^ Fenway Health, Boston, Massachusetts, USA.; ^4^ Ariadne Labs, Boston, Massachusetts, USA.

**Keywords:** patient–provider communication, pregnancy, prenatal care, digital health equity, digital health literacy

## Abstract

**Background:**

Patient–provider communication (PPC) increasingly occurs in online patient portals. Variations in portal usage might worsen communication inequities for pregnant people of color (POC), widening the digital divide. The objective of this study was to examine the relationships between respectful PPC, patient portal usage, and digital health literacy (DHL) in pregnant POC.

**Methods:**

A multimethod cross-sectional survey design was used. Ordered logistic regression was performed to determine the relationship between PPC and portal use, controlling for trimester prenatal care was initiated, insurance type, age, gestational age, and parity. The moderating effect of Digital Health Literacy Instrument (DHLI) was tested on the association between portal usage and PPC.

**Results:**

A total of 130 self-identified pregnant POC participated in the study. Participants who did not use the portal had 68% lower odds of rating higher quality PPC (odds ratio [OR] = 0.32, 95% confidence interval [CI] = 0.12–0.86, *p* = 0.02). Participants with public versus private insurance had 62% lower odds of rating high-quality PPC (OR = 0.38, 95% CI = 0.14–0.99, *p* = 0.04). For portal users, DHL moderated the association between PPC and portal use - eHealth Literacy Scale (adjusted OR [aOR] = 1.06, 95% CI = 1.01–1.12, *p* = 0.02) and DHLI (aOR = 2.36, 95% CI = 1.12–4.95, *p* = 0.02). The moderation effect of DHLI was also significant among limited portal users (aOR = 2.32, 95% CI = 1.04–5.19, *p* = 0.04).

**Conclusion:**

Addressing the digital divide for pregnant POC requires further investigation into portal non-users with consideration to insurance type, DHL, and social determinants of health.

**Figure:**
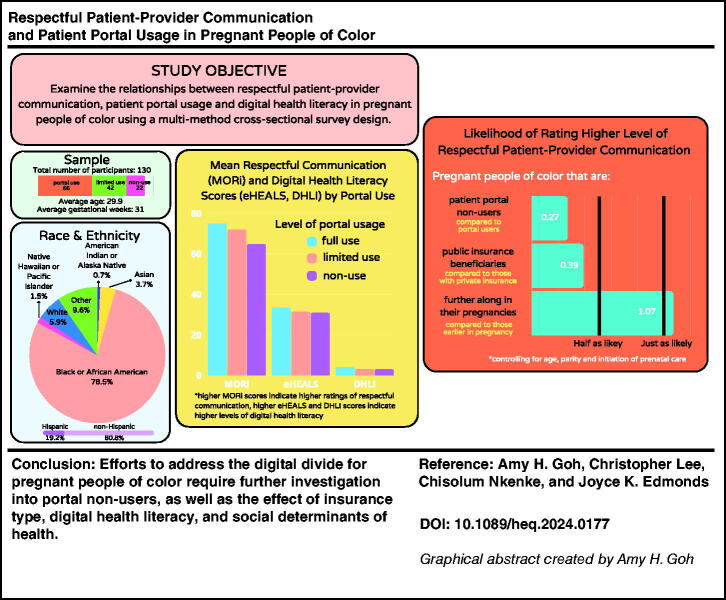


## Introduction

Patient–provider communication (PPC) and digital communication are public health priorities in the United States. Healthy People 2030 goals include improving the quality of communication between providers and patients, including face-to-face and digital communication.^1^ Multiple studies have shown that pregnant people report difficulty in communicating with their prenatal providers.^2,3^ Pregnant people of color (POC) have cited racism, discrimination, feeling unheard, and disrespect as key issues contributing to poor communication with health care providers.^4–6^ Respectful PPC communication is defined by the birthing person feeling comfortable and empowered to accept or decline care; it is the absence of discrimination and behavior change due to the potential of feeling disrespected.^7^ Racial inequities in communication may play a significant role in the pronounced differences in perinatal morbidity and mortality in pregnant POC, especially among Black pregnant people.^8–10^

Despite relevant Healthy People goals and government initiatives focused on patient portal (portal) access,^11^ limited studies exist on portal use during pregnancy. Overall, 39 − 72% of pregnant patients enroll in portals such as Epic MyChart® (MyChart).^12,13^ However, researchers have shown that non-Hispanic Black and Hispanic pregnant people are less likely to utilize portals.^14,15^ Pregnant people who are diagnosed as high risk are less likely to utilize portals,^13,14,16^ whereas factors associated with higher portal usage include older age, first-time parenthood, marital status, starting prenatal care earlier, and having more prescriptions at the initial prenatal visit.^13–15^

Disparities in portal usage could exacerbate the digital divide in pregnancy, with digital health literacy (DHL) being a contributing factor.^17–19^ DHL involves the ability to find, assess, apply, and communicate information about one’s health through online and digital health tools.^20,21^ Few studies have examined DHL during pregnancy in the United States.^22–24^ Research in other specialties demonstrates that people with higher DHL and increased portal use rate have higher quality and satisfaction with care^25,26^; however, this relationship during pregnancy has not been explored.

Our objective was to examine the relationships between respectful PPC, portal usage, and DHL in pregnant POC. We hypothesized that portal use would be significantly associated with positive perceptions of PPC and that greater DHL would positively moderate the relationship between PPC and portal usage.

### Positionality

We recognize that lived experiences and privilege influence how research is conducted, and therefore, we present our positionalities. A.H.G. is a cisgender woman, Asian American midwife and researcher in the U.S. Northeast. C.L. is a cisgender male and a White endowed professor at a university in Massachusetts. C.N. is a cisgender woman and an African public health practitioner in the United States. J.K.E. is a cisgender woman and a White nurse scientist in Massachusetts.

## Methods

### Study Design

Results reported herein represent the primary quantitative aim of a multimethod cross-sectional survey study. The study was approved by the institutional review boards of Boston College and Boston Medical Center.

### Sample and Data Collection

The sample was composed of pregnant POC receiving care from a prenatal clinic within a safety net hospital in Boston between June and September 2023. Inclusion criteria included the following: (1) self-identified person of color, (2) receiving prenatal care at the hospital’s urban clinic with a certified nurse-midwife, obstetrician, or family medicine physician, (3) at least 20 weeks pregnant, (4) at least 18 years of age, and (5) fluency in English. Participants were excluded if they were unable to understand the consent process. We recruited participants by convenience sampling. Participants who provided informed consent completed the survey in person at the prenatal clinic using study-approved iPads, personal cellphones, or computers. Participants were provided a $30 gift card after survey completion.

### Measurements

#### Demographic data

The following demographic data were collected: age, race, ethnicity, parity, initiation of prenatal care, and insurance type. These variables were selected based on previous research on portal usage in pregnancy.^12–15^ Race and ethnicity were categorized according to the National Institute of Health Policy and Guidelines on the Inclusion of Women and Minorities as Subjects in Clinical Research from 2023.^27^ Participants could self-identify their race and ethnicity in addition to selecting from categories. Parity was categorized as nulliparous or multiparous. Initiation of prenatal care was categorized as first, second, or third trimester. Insurance type was based on the Massachusetts birth certificate data categories of private, public, or self-pay/free care.^28^

#### Mothers on Respect Index

**FIG. 1.**
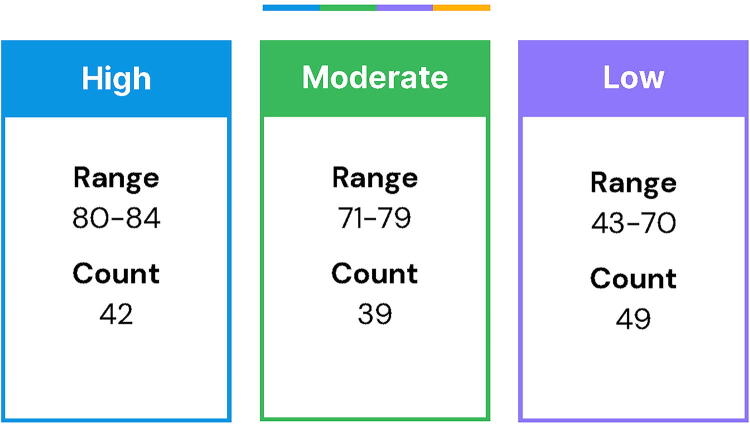
Mothers on Respect Index scores divided into tertiles.

The Mothers on Respect Index (MORi) was used to measure respectful PPC.^7^ MORi is a 14-item survey developed through community-led participatory action research involving pregnant people, community members, and researchers.^7^ MORi was content validated in a racially diverse sample in the United States and had a Cronbach’s α of 0.94.^29^ The unidimensional MORi score is calculated by summing all item scores. Previous researchers have categorized MORi scores to better understand the experience of perinatal care.^30,31^ Figure 1 outlines how we categorized MORi scores into tertiles based on sample distribution; respectful communication categories were defined as low (42–70), moderate (71–79), and high (80–84).

#### Patient portal usage

A standardized measure of portal usage does not exist; however, our study used two of the most common measures: *adoption* and *intensity.*^32^ *Adoption* is defined as whether the patient has registered or logged on to use the portal, and *intensity* is defined as the extent to which participants utilize the portal.^32^ Several studies on POC have included these two measures.^33–35^ We measured *adoption* by asking whether participants utilize the portal and to confirm whether they are users or non-users of the portal. We measured *intensity* by asking participants if they had sent a secure message through the portal during pregnancy. We developed three categories of portal use: (1) portal use or participants who sent a message in MyChart; (2) portal limited use or participants who used MyChart but did not send a message; and (3) portal non-use or those who did not use MyChart. We also asked six exploratory questions about how often various sections of MyChart were used and four questions about internet use. These survey questions were adapted from the Health Information National Trends Survey.^36^

#### Digital Health Literacy Instrument

**FIG. 2.**
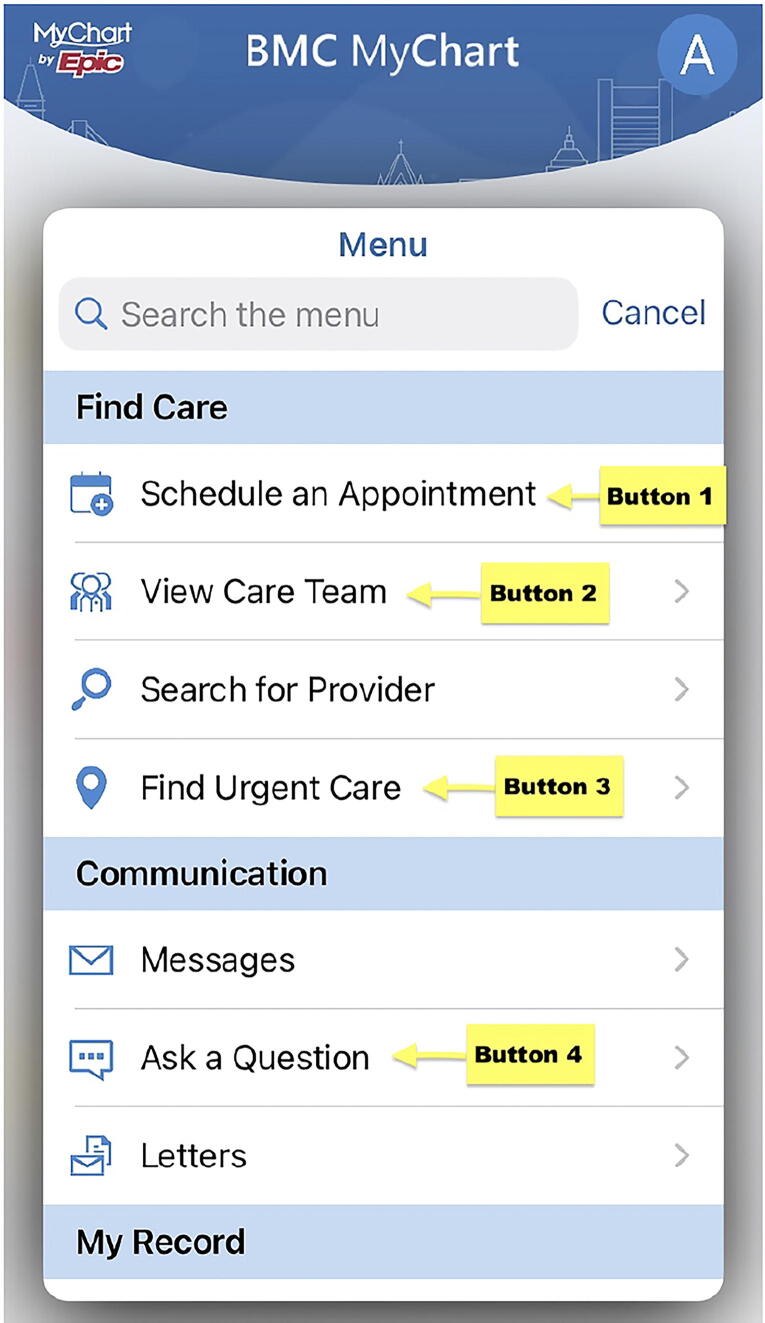
Snapshot of adapted Digital Health Literacy Skills Test.

The Digital Health Literacy Instrument (DHLI) was used to assess DHL.^21^ The DHLI is a 21-item self-reported scale that has been adapted to U.S. audiences^37^; however, DHLI has never been validated in pregnant people. Based on previous studies,^38^ we reported the unidimensional DHLI score as the average of the sum of scores from each of the six categories: (1) operational skills, to use the computer and internet browser; (2) navigation skills, to navigate and orientate on the web; (3) information searching skills, to use correct search strategies; (4) evaluating reliability and relevance of online information; (5) adding self-generated content to web-based apps; and (6) protecting and respecting privacy while using the internet. The maximum score is 4, indicating the highest level of DHL. Cronbach’s α was 0.87 for the DHLI.^21^ The DHLI offers a skills test (Fig. 2), which was adapted to the pregnant population. Our skills test consisted of six multiple-choice questions and one free text question (Table 1).

**Table 1.** Results of the Digital Health Literacy Index Skills Test

Digital health literacy skill	Total *n* = 130 *n* (%)	Portal use *n* = 66 *n* (%)	Portal limited use *n* = 42 *n* (%)	Portal non-use *n* = 22 *n* (%)	$x^{2}$	*p*
Percentage of participants able to correctly:						
Locate the minimize button	80 (62.5)	49 (61.3)	23 (28.8)	8 (10)	2.86	0.24
Locate the back tab	81 (63.3)	46 (56.8)	24 (29.6)	11 (13.6)	0.81	0.67
Locate on webpage where to click for desired information	113 (87.5)	62 (54.9)	33 (29.2)	17 (15.0)	0.51	0.77
Locate where to click to find provider’s name on MyChart	101 (78.3)	57 (56.4)	31 (30.7)	13 (12.9)	1.00	0.60
Select which website will provide most reliable and accurate information	34 (26.2)	20 (58.8)	13 (38.2)	1 (0.03)	4.37^a^	0.11
Select from forum members who maintained the most privacy	68 (52.3)	38 (55.9)	21 (30.9)	9 (13.2)	0.64	0.73

^a^ Represents *x*^2^ calculated with cell value <5.

#### eHealth Literacy Scale

Because the DHLI has not been validated in pregnancy, the eHealth Literacy Scale (eHEALS)^20^ was used as an alternative measure of DHL. The eHEALS was developed in 2006 and is a unidimensional eight-item scale to measure an individual’s searching, knowledge, and evaluation of electronic health information.^20^ The eHEALS score is calculated by adding the scores of the eight questions. The range of possible scores is 8–40, with a higher score signifying higher eHealth literacy. Cronbach’s α for the eHEALS was 0.88.^20^

### Data analysis

Statistical analysis was performed using Stata version 18.^39^ Using ordered regression modeling of PPC (MORi) to examine the impact of variables such as portal use, age, gestational age, race, ethnicity, parity, health insurance, and initiation of prenatal care, we estimated a required sample size of 130 participants. Analysis with power of 0.80, α of 0.50, estimated effect size of 0.15, and anticipated attrition rate of up to 15% indicated that a total of 130 participants would be sufficient. Descriptive statistics, including counts, frequencies, measures of central tendency, and dispersion, were used to describe the sample. One-way analysis of variance (ANOVA) tests were performed to identify differences in demographic and DHL scores among the categories of MORI scores. Ordered logistic regression was used to model PPC as the dependent variable, with odds ratios (ORs) >1 indicating higher quality PPC. First, an unadjusted model was constructed with portal usage as the single independent variable. Next, this model was refined to adjust for the influence of study covariates.^12–14^ We anticipated that the odds of higher quality PPC based on portal use would remain significant after controlling for the covariates. The last model was developed to test the moderating role of DHL on the relationship between portal usage and PPC. The interaction term (*DHL*  × *portal usage*) was entered into both the unadjusted and adjusted models. We anticipated that greater DHL would positively moderate the relationship between portal usage and PPC. Results of ordered regression modeling are presented in ORs, 95% confidence intervals (95% CIs), and their respective *p* values.

## Results

Table 2 summarizes the demographic characteristics and internet use of the 130 participants in our sample. The average age of participants was 29.9 years, and gestational age was 31 weeks. Most participants were multiparous (62.3%), insured by public insurance (85.4%), received care from a certified nurse-midwife (61.5%), and initiated prenatal care in the first trimester of pregnancy (68.5%).

**Table 2.** Sample Characteristics

	Total*n* (%)	Portal use*n* (%)	Portal limited use*n* (%)	Portal nonuse*n* (%)
Parity				
Nulliparous	49 (37.3)	29 (59.2)	15 (30.6)	5 (10.2)
Multiparous	81 (62.3)	37 (45.7)	27 (33.3)	17 (21.0)
Race				
American Indian or Alaska Native	1 (0.7)	0	1 (100)	0
Asian	5 (3.8)	2 (40)	3 (60)	0
Black or African American	106 (81.5)	54 (50.9)	31 (29.2)	18 (17.0)
Native Hawaiian or Pacific Islander	2 (1.5)	1 (50)	0	1 (50)
White	8 (6.2)	5 (62.5)	3 (37.5)	0
Other	13 (10)	6 (46.2)	4 (30.8)	3 (23.1)
Ethnicity				
Hispanic	25 (19.2)	12 (48)	10 (40)	3 (12)
Insurance				
Public	111 (85.4)	52 (46.8)	38 (34.2)	21 (18.9)
Private	19 (14.6)	14 (73.7)	4 (21.1)	1 (0.1)
Self-pay	0	0	0	0
Provider type				
Midwife	80 (61.5)	43 (53.8)	26 (32.5)	11 (13.8)
Obstetrician	49 (37.7)	22 (44.9)	16 (32.7)	11 (22.4)
Family medicine physician	1 (0.77)	1 (100)	0	0
Initiation of prenatal care				
First trimester	89 (68.5)	48 (53.9)	30 (33.7)	11 (12.4)
Second trimester	32 (24.6)	17 (53.1)	7 (21.9)	9 (28.1)
Third trimester	9 (6.9)	2 (22.2)	5 (55.6)	2 (22.2)
Do you ever go online to access internet?^a^				
No	14 (10.8)	3 (21.4)	5 (35.7)	6 (42.9)
Barriers to internet use^b^				
Bad internet connection through phone	7 (5.4)	2 (28.6)	3 (42.9)	2 (28.6)
Do not have computer/laptop at home	5 (3.8)	1 (20)	2 (40)	2 (40)
Can’t pay for internet	1 (0.8)	0	1 (100)	0
Do not like using the internet	1 (0.8)	0	0	1 (100)
Do not know how to use the internet	1 (0.8)	0	0	1 (100)
How do you connect to the internet?^b^				
Cellular network	83 (63.9)	47 (56.6)	26 (31.3)	10 (12.0)
Wireless connection (Wi-Fi)	69 (53.1)	38 (55.1)	24 (34.8)	7 (10.1)
Broadband	5 (3.9)	3 (60)	1 (20)	1 (20)
Dial-up	4 (3.1)	1 (25)	2 (50)	1 (25)
Don’t know	8 (6.2)	3 (37.5)	2 (25)	3 (37.5)
Daily use of cellphone for internet	116 (92.9)	62 (53.4)	36 (31.0)	18 (15.5)
Daily use of computer at work for internet	45 (38.8)	25 (55.6)	14 (31.1)	6 (13.3)
Daily use of computer at home for internet	31 (25.6)	15 (48.4)	11 (35.5)	5 (16.1)
Never used computer in public place (library)	65 (57.0)	36 (55.4)	18 (27.7)	11 (16.9)

*N* = 130 (*n* = 66 for portal users, *n* = 42 for portal limited users, *n* = 22 for portal nonusers). Participants were on average 29.9 years old (*SD* = 6.1) and 31 weeks gestation (*SD* = 5.3). Participant and gestational age (did not differ by portal use).

^a^ Reflects the number and percentage of participants answering “no” to this question.

^b^ This item had a “select all that apply” option.

SD, standard deviation.

Most participants identified as Black or African American (81.5%), and those who opted to further identify within this category were Haitian (*n* = 6), Cape Verdean (*n* = 5), Caribbean American (*n* = 2), Ethiopian (*n* = 2), East Indian (*n* = 1), Ugandan (*n* = 1), and Mixed Race (*n* = 1). In the Asian category (3.8%), participants who further self-identified were Pakistani (*n* = 1) and Filipino (*n* = 1). In the White category (6.2%), two participants further self-identified as Hispanic. In the Other race category (10%), participants self-identified as Spanish (*n* = 1), Latina (*n* = 1), and Hispanic (*n* = 1). American Indian or Alaska Natives consisted of 0.7% of the sample, and Native Hawaiian or Pacific Islander was 1.5% of the sample. Those who identified as Hispanic were 19.2% of the sample.

An ANOVA test revealed a significant effect of PPC on the three categories of MORi scores, on DHLI scores (*F*_2, 127_ = 4.37, *p* = 0.015), and on eHEALS scores (*F*_2, 127_ = 3.90, *p* = 0.023). Further ANOVA testing indicated a significant effect of the three categories of portal use on DHLI scores (*F*_2, 127_ = 4.27, *p* = 0.016), but not on eHEALS scores (*F*_2, 127_ = 2.18, *p =* 0.118).

We used ordered logistic regression to create the unadjusted model (Table 3) and found that participants portal non-users had significantly lower odds of rating higher quality PPC compared with portal users and limited users (OR = 0.27, 95% CI = 0.11–0.68, *p* = 0.01). Findings were not significant for limited users (OR = 0.73, 95% CI = 0.36–1.51, *p* = 0.40). In the adjusted model, portal non-users had significantly lower odds of rating higher quality PPC compared with portal users and limited users (OR = 0.32, 95% CI = 0.12–0.86, *p* = 0.02). Findings were not significant for limited users (OR = 0.78, 95% CI = 0.37–1.65, *p* = 0.52). Compared with participants with private insurance, those with public insurance had significantly lower odds of rating higher quality PPC, holding all other variables constant (OR = 0.38, 95% CI = 0.14–0.99, *p* = 0.04). For post-estimation tests, the likelihood-ratio test was performed and indicated no violation of the proportional odds assumption (*p* = 0.16).

**Table 3.** Summary of Ordered Logistical Regression Model: Patient–Provider Communication, Portal Use, and Covariates

PPC	OR	95% CI		*p*	aOR^a^	95% CI		*p*
		LL	UL			LL	UL	
Portal use								
Limited portal use	0.73	0.36	1.51	0.40	0.78	0.37	1.65	0.51
Portal no use	0.27	0.11	0.68	0.01^*^	0.32	0.12	0.86	0.02^*^
Trimester-initiated prenatal care								
Second trimester	0.66	0.31	1.38	0.27	0.85	0.39	1.85	0.68
Third trimester	1.70	0.48	6.00	0.41	2.05	0.53	7.55	0.30
Public insurance	0.39	0.16	0.97	0.04^*^	0.38	0.14	0.99	0.04^*^
Age	1.00	0.95	1.05	0.96	0.99	0.93	1.05	0.07
Gestational age	1.07	1.00	1.13	0.04^*^	1.06	0.99	1.13	0.12
Multiparous	0.88	0.46	1.67	0.69	1.17	0.57	2.39	0.67

^a^ The model was adjusted for age, gestational age, parity, insurance type, and initiation of prenatal care.

^*^ *p* < 0.05.

aOR, adjusted odds ratio; CI, confidence interval; LL, lower limit; OR, odds ratio; PPC, patient–provider communication; UL, upper limit.

To understand the moderating effect of DHL on the relationship between PPC (MORi) and portal use, first, we ran an unadjusted model demonstrating the independent effects of DHL and portal use on ratings of PPC (Table 4). Controlling for portal use, each additional point on both DHL scales was associated with higher odds of rating high-quality PPC versus moderate and low quality—eHEALS (OR = 1.06, 95% CI = 1.00–1.11, *p* = 0.04) and DHLI (OR = 2.37, 95% CI =1.10–5.10, *p* = 0.03). Controlling for DHL, compared with limited users, portal non-users had lower odds of rating high-quality PPC versus moderate and low quality—eHEALS (OR = 0.29, 95% CI = 0.11–0.75, *p* = 0.01) and DHLI (OR = 0.31, 95% CI = 0.12–0.81, *p* = 0.02). In the unadjusted moderation model, the moderation effect was significant for both tools at the level of portal users—eHEALS (OR = 1.06, 95% CI = 1.01–1.14, *p* = 0.02) and DHLI (OR = 2.54, 95% CI = 1.11–5.41, *p* = 0.01). The moderation effect of DHLI was also significant at the level of limited users (OR = 2.45, 95% CI = 1.11–5.41, *p* = 0.03).

**Table 4.** Summary of Moderation Analysis of Digital Health Literacy and Portal Use on Ratings of Patient–Provider Communication

PPC	OR	95% CI		*p*	aOR^a^	95% CI		*p*
		LL	UL			LL	UL	
eHEALS	1.06	1.00	1.11	0.02^*^				
eHEALS × portal use								
Portal use	1.06	1.01	1.12	0.02^*^	1.06	1.01	1.12	0.02^*^
Portal limited use	1.06	1.00	1.12	0.05	1.06	1.00	1.12	0.06
Portal no use	1.02	0.97	1.08	0.42	1.03	0.97	1.09	0.36
DHLI	2.37	1.10	5.10	0.03^*^				
DHLI × portal use								
Portal use	2.54	1.11	5.41	0.01^*^	2.36	1.12	4.95	0.02^*^
Portal limited use	2.45	1.11	5.41	0.03^*^	2.32	1.04	5.19	0.04^*^
Portal no use	1.80	0.82	3.96	0.14	1.78	0.79	4.00	0.16

^a^ The model was adjusted for age, gestational age, parity, insurance type, and initiation of prenatal care.

^*^ *p* < 0.05.

DHLI, Digital Health Literacy Instrument; eHEALS, eHealth Literacy Scale.

**FIG. 3.**
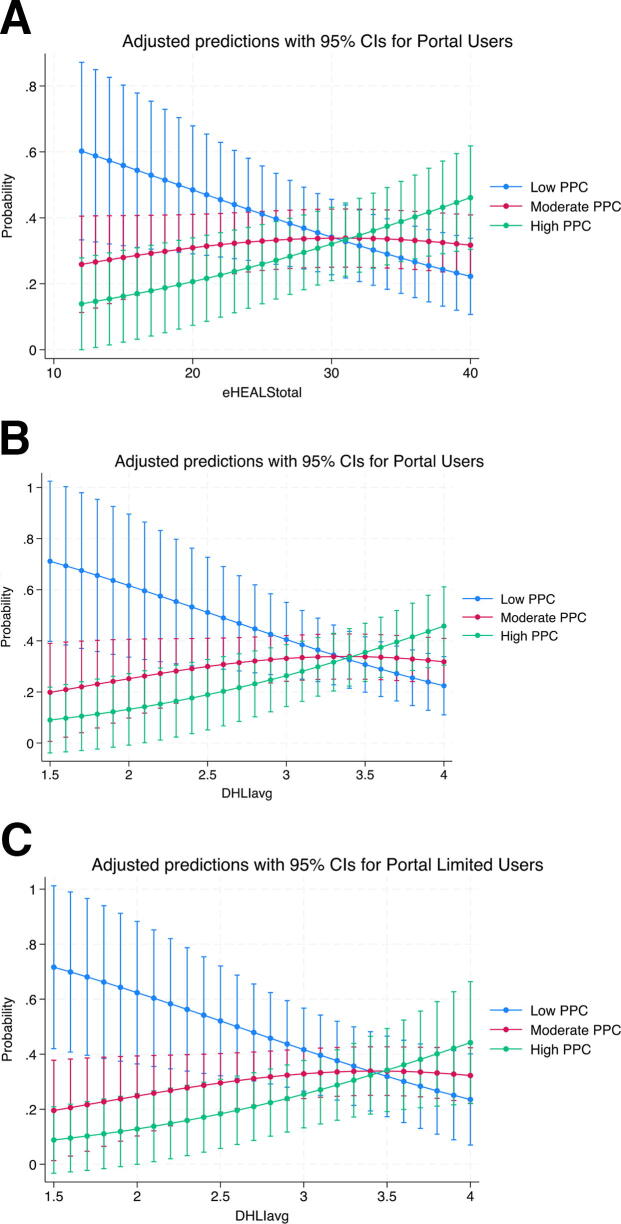
Marginal effects of the significant moderation models of digital health literacy. Adjusted predictions with 95% CIs for portal users—eHealth Literacy Scale. CI, confidence interval; PPC, patient–provider communication.

In the adjusted moderation model, controlling for age, gestational age, parity, initiation of prenatal care, and insurance type, the moderation effect was significant for both tools at the level of portal users, eHEALS (OR = 1.06, 95% CI = 1.01–1.12, *p* = 0.02) and DHLI (OR = 2.36, 95% CI = 1.12–4.95, *p =* 0.02). The moderation effect of DHLI was also significant at the level of limited users, controlling for all variables (OR = 2.32, 95% CI = 1.04–5.19, *p* = 0.04). Plotting the marginal effects revealed that the marginal effect of DHLI increased for those who rated the highest level of PPC in both portal users and non-users (Fig. 3). However, the effect was the opposite for those who rated the lowest level of PPC, with the marginal effects of DHLI decreasing. For those with moderate ratings of PPC, the marginal effect of DHLI initially increased but then slightly decreased.

## Discussion

Our study examined the relationship between PPC and three levels of portal usage among pregnant POC. We found that participants who did not use the portal had lower odds of rating higher PPC compared to participants who used the portal. Similarly, participants covered by public insurance had lower odds of rating higher PPC compared with those covered by private insurance. These findings demonstrate an inequity in the quality of PPC between users and non-users of the portal among a sample of pregnant POC.

Research on provider trust among pregnant POC provides insight into our findings. Systematic and interpersonal racism and discrimination lead to mistrust in providers and health care institutions.^40–42^ A qualitative study on pregnancy and birthing experiences of Asian American, Black, Latina, Middle Eastern, and Pacific Islander women found that mistrust in providers led to little engagement with prenatal care.^3^ Though portal non-users report privacy concerns,^43^ further exploration into the relationship between medical mistrust and portal engagement in pregnancy is needed.

Our findings might also be explained by the digital divide, which is known to operate on three levels. The first level is marked by inequities between people who have and do not have access to broadband internet. The second level pertains to differences in digital skills, and the third level involves the beneficial outcomes of digital usage. Most participants in our sample utilized the internet; however, the highest proportion of people who did not use the internet was among portal non-users, which relates to the first level of the digital divide. The second level of the digital divide was also evident through portal non-users, compared with users and limited users, having the lowest DHL scores. Portal nonusers had a lower odds of rating high levels of PPC compared with the other two categories of users, reflective of the third level of the digital divide.

We found that DHL had a moderating effect on the relationship between portal use and PPC. We found that the marginal effects of DHL for portal users and non-users were highest for those who rated the highest levels of PPC and that DHL had the lowest effect for those who had low PPC ratings. DHL is known as a super determinant of health due to its ability to measure social determinants of health that may manifest through tasks that require DHL.^44^ Though there is a paucity of research on the relationship between DHL and PPC, multiple studies have demonstrated the positive relationship between health literacy and PPC.^45,46^ As research has demonstrated the strong relationship between health literacy and DHL,^21^ future studies should examine the directionality of the relationship between PPC and DHL in pregnancy.

Though few research studies have examined DHL in pregnancy,^47^ one cohort study of 10,038 nulliparous birthing people from eight major medical centers in the United States found that those with inadequate health literacy had higher odds of cesarean birth and other adverse outcomes.^48^ Considering our finding that DHL was a moderator in the relationship between PPC and portal use, further exploring how DHL affects perinatal outcomes in pregnant POC, may contribute to strategies to help bridge the digital divide. We found that participants with private insurance coverage had a higher percentage of portal users who sent messages to their providers compared with participants with public insurance. This finding is consistent with those from researchers who have found that pregnant people with public insurance were less likely to enroll in portals.^13,15^ Our study outlined digital health inequity by insurance type in pregnant POC; however, numerous studies have demonstrated adverse perinatal outcomes in pregnant POC, particularly Black and Native American birthing people, with public insurance.^49,50^ Future research should consider the effects of portal usage for pregnant POC who experience discrimination due to race, insurance type, and other factors.

### Strengths and limitations

This is the first study on pregnant people that has assessed DHL using the DHLI. The cross-sectional study design and convenience sample limit generalizability and causal inference. Given the small sample size of some of the racial groups, there may have been variations within race and ethnicity that were missed; disaggregating these data in future studies is needed. All participants in the study were English speaking, which may not have reflected the responses of pregnant people whose primary language is not English. The eHEALS is limited in its ability to measure elements such as engagement with providers via digital tools. Limitations of the DHLI include minimal studies that have evaluated the tool.^51^

## Conclusion

Our findings indicate that pregnant POC who are non-users of the patient portal are more likely to have lower ratings of communication with their prenatal providers compared with those who use the patient portal. As policies such as the Bipartisan Infrastructure Deal and the 21st Century Cures Act^52^ enhance accessibility to the internet and online medical records among patients, it is important to assess the needs of pregnant POC who are not utilizing the internet. Asking about communication preferences and assessing DHL at the initial prenatal visit, or at minimum, evaluating skills to access and navigate the patient portal, can provide valuable insights into patients’ communication needs. This approach will support quality PPC for pregnant POC and help address digital health inequities during pregnancy.

## Abbreviations Used

DHL: Digital health literacy
DHLI: Digital Health Literacy Instrument
eHEALS: eHealth Literacy Scale
MORi: Mothers on Respect Index
POC: People of color
PPC: Patient-provider communication
